# Structure-based virtual screening and molecular docking for the identification of potential novel EGFRkinase inhibitors against ovarian cancer

**DOI:** 10.6026/97320630015287

**Published:** 2019-04-15

**Authors:** Khalid Hussain Wali Sait, Qamre Alam, Nisrin Anfinan, Othman Al-Ghamdi, Arshi Malik, Rana Noor, Farheen Jahan, Mohammed Tarique

**Affiliations:** 1 Department of Obstetrics and Gynecology, Gynecology Oncology Unite, Faculty of Medicine, King Abdulaziz University, Jeddah, SaudiArabia; 2 King Fahd Medical Research Center, King Abdulaziz University, Jeddah. Saudi Arabia; 3 Department of Obstetrics andGynecology, Gynecology Oncology Unite, Faculty of Medicine, King Abdulaziz University, Jeddah, Saudi Arabia; 4 Department of Biological Sciences, Faculty of Science, University of Jeddah, Kingdom of Saudi Arabia; 5 Department of Clinical Biochemistry, College ofMedicine, King Khalid University, Abha, Saudi Arabia; 6 Department of Biochemistry, Faculty of Dentistry, Jamia Millia Islamia, JamiaNagar, New Delhi-110025, India; 7 Department of Biosciences Jamia Millia Islamia, Jamia Nagar, New Delhi-110025, India; 8 8Center forInterdisciplinary Research in Basic Sciences, Jamia Millia Islamia, Jamia Nagar, New Delhi-110025, India

**Keywords:** ADMET, Biological activity spectrum, EGFR tyrosine kinase, Pharmacokinetic, Inhibitor

## Abstract

Epidermal Growth Factor Receptor (EGFR) is, for the most part, deregulated and over-communicated in ovarian disease, which is legitimately connected with STAT3 enactment that prompts the collection of hostile to apoptotic occasions and along these lines, docetaxel medicate obstruction happens. As to, expanding of docetaxel medicate affectability by focusing on EGFR receptor alongside docetaxel drugs is one of the real techniques in ovarian disease treatment. In this specific circumstance, utilizing atomic recreation considers, the present examination depicted the auxiliary and pragmatic properties of IBS Database mixes as a potential inhibitor of EGFR tyrosine kinase, and furthermore ADMET had researched its Pharmacokinetic profile. As indicated by the outcomes, STOCK1N-98911, STOCK1N- 98869, and STOCK1N-98896 have appeared tremendous restricting vitality by associating with critical build ups in the dynamic site. Natural movement range forecast of these mixes indicated potential anticancer properties by demonstrating important collaboration with EGFR tyrosine kinase. Besides, the investigation is likewise valuable for further clinical based examinations and furthermore for the approval of toxicological and pharmacokinetic contemplate

## Background

 Recently, the primary source of death in ladies is ovarian malignancy adding up to 1, 40,000 passings consistently 1. Even though most ladies with epithelial ovarian malignant growth (EOC) are analyzed at cutting edge organizes, the reduced expectation of this malady causes more passing 2. Thus, the clinical reaction rate at the start is extremely high, where platinum-based mixes are benchmark first-line operators for ovarian malignant growth treatment.^3^. Be that as it may, because of the advancement of obtained chemo resistance in most ovarian malignant growth, the treatment procedures are presently in a significant test. 4,5. In this manner, in such condition, there is a pressing need to create novel and useful helpful systems against malignant ovarian growth 6,7. Epidermal Growth Factor Receptor (EGFR), which has a place with the group of Human Epidermal Receptor (HER), is very communicated in numerous fatalities, basically in ovarian disease 8. It's one of the tyrosine kinases and for the most part initiated by extracellular ligands that trigger receptor auto phosphorylation, which may prompt the actuation of downstream pathways engaged with expansion, survival, angiogenesis, and attack 9, 10. Ongoing investigations have demonstrated that the EGFR pathway is intermittently enacted in most malignant growth cell, and it is focused on restraint with a little atom kinase inhibitor has been useful for lung, bosom and ovarian disease with EGFR transformations 11, 12. Along these lines, the present examination was meant to explore the helpful properties of IBS information base as EGFR tyrosine kinase inhibitor through different computational investigation, including atomic docking, ADME/T examination, and organic range 13, 14. 

## Methodology

### Identification of EGFR gene:

All the sequences of the EGFR were retrieved from the genome database NCBI (http://www.ncbi.nlm.nih.gov). The FASTA formats of the retrieved sequences were used for BLAST-P analysis. The series was downloaded and analyzed in detail. The nucleotide sequence of EGFR is 3630 bases, and it codes for a protein of 1210 amino acids. Multiple-sequence alignment was done by using ClustalW (http://www.ebi.ac.uk/Tools/msa/clustalw2/) and Clustal Omega (http://www.ebi.ac.uk/Tools/msa/clustalo/). 

### Domain Organization:

The retrieved sequences were in-silico studied, and various domains were manually assigned and confirmed by using Pfam, Prosite and integrated software, InterProScan. And conserved motifs were identified manually as well as using Pfam and InterProScan software. 

### Selection of Ligands and protein molecule:

Schematic outline of the work process examine configuration is shown in Figure 3. Ligand atoms were chosen from IBS natural compound library (InterBioScreen Ltd). Ligands were set up by utilizing the LigPrep module of the Maestro 10.5 application. LigPrep performs numerous amendments on the ligands, for example, the expansion of hydrogen, 2D - 3D transformation, rectified bond lengths and bond angles, low-vitality structure, and ring compliance. Separated from that another parameter, for example, ionization does not change, tautomers not made and hold specific philanthropies create at most one for every ligand were utilized as a default parameter in Maestro 10.5. After that all iota compel, and particle types were appointed by the optimized potential for fluid reproductions (OPLS_2005) drive field 15, 16. At long last, one adaptation for every ligand was generated, and ligands are prepared for docking. The structure of the epidermal growth factor receptor (EGFR) kinase space (PDB id: 1M17) is recovered from the protein information bank. Maestro 10.5 protein arrangement wizard applications were performed for the redress of crude structure, where changes, for example, expansion of hydrogen atoms, doling out security orders, a formation of zerorequest bonds to metal, making of disulfide securities, fixing of the charges and introduction of gatherings were consolidated into the fibrous structure. 

### Molecular docking:

Molecular docking studies using the selected ligand molecules were conducted using Maestro 10.5 molecular docking suite 17, 18. Every one of these mixes was docked into target protein as needs be with positions, introductions, and compliances of the ligand in the receptor restricting site, and the docking structure having the most reduced vitality was favored. In the present examination, we screened around 50,000 standard mixes from the IBS againstEGFR kinase. IBS characteristic mixes docked with each chosen protein particles by utilizing HTVS. To give a superior connection be tween's correct stances and high scores, GLIDE-XP mode was used accordingly on the adaptations picked from HTVS mode. Based on Score, we select 20 mixes for GLIDEXP atomic docking. After the fruition of ligands and proteins planning, a receptor network record was created. For running the framework age module, we have scaled van der Waal radii of receptor molecules by 1.00 Å as the default setting of Maestro 10.5. The dynamic site of the receptor keeps up an exact scoring capacity with thermodynamically most ideal vitality and is determined on a framework by different arrangements of fields. After the method of the receptor network document, adaptable ligands with rigid receptor-based sub-atomic docking were performed. The best-fit mixes have been decided for each objective by ideal vitality esteem and kinds of communications. 

### Prime MM-GBSA:

Prime MM-GBSA approach was utilized to compute ligand restricting energies and ligand strain energies for a ligand and a single receptor. MM-GBSA is a strategy that joins OPLSAA atomic mechanics energies (EMM), an SGB solvation display for polar solvation (GSGB), and a non-polar solvation term (GNP) made out of the non-polar dissolvable available surface region and van der Waals communications. Here, the Glide present watcher document of the best conformation picked was given as the source in Prime MM-GBS A simulation. The all-out free vitality of official: ΔGbind = G-complex - (G-protein + Gligand), where G = EMM + GSGB + GNP 

### Absorption, distribution, metabolism, excretion, and toxicity (ADME/T) properties studies:

 Most of the medication competitors don't prevail in clinical trials because of poor toxicology appraisals (ADME/T). In this way, ADME/T properties of best-docked mixes were anticipated utilizing QikProp use of Maestro 10.5 (auxiliary, physicochemical, biochemical, pharmacokinetics, and harmfulness properties). It predicts characteristic features of the atoms (medicate like properties, for example, octanol/water segment, log BB, by and substantial CNS action, log IC50 for Herg K+ channel blockage, Caco-2, MDCK cell porousness and log Khsa for human serum egg whites official 19, 20. 

### Boiled-egg Plot:

 A boiled-Egg plot loan consoling help and gives an interesting measurable plot to help the two aloof expectations made, which is gastrointestinal retention and cerebrum entrance of little particles, which is necessary for revelation, and improvement of medications. Both the parameters are spoken to on a cartesian plane in the state of obscurations and incorporate other vital parameters, for example, MW, TPSA, MLOGP, GI, and BBB to recondition the boiled Egg plot. As needs are, in the cartesian plane, if our mixes rest in the yolk locale spoken to by the yellow oval, the likelihood of BBB-Blood Brain Barrier is though if the blends relax under white zones, the guess of gastrointestinal retention is enhanced. Adjacent to these locales, if the mixes rest in dark areas barring the "egg" or are out of the scope of the chart, the combinations are nonabsorptive even non-mind infiltration, and consequently, it mulled over as a commented box. The areas are not selective of one another. 

### Biological activity spectrum (BAS):

BAS of a best-docked compound speaks to the complex of pharmacological impacts and the characteristic properties of the compound relied upon its essential feature. The pharmacological impacts, displayed by a mixture and its correspondence with organic substances were anticipated by PASS online by transferring the SMILES string of regular mixes 21. 

## Results and Discussion

 In this study we have reported the in silico analysis of all the EGFR gene of ovarian cancer. 

### Identification and sequence analysis EGFR gene:

**Figure 1:**
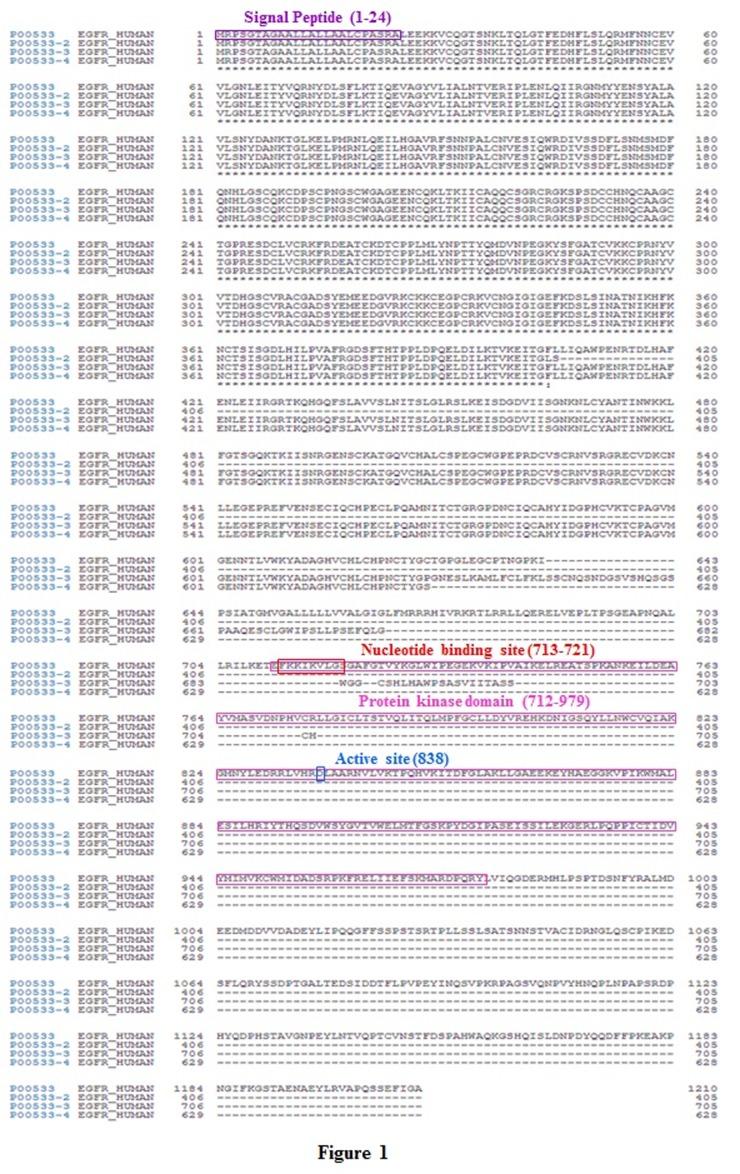
Multiple sequence alignment of EGFR. Comparison of amino acid sequences for EGFR is shown. The alignment was done using BLAST program (http://blast.ncbi.nlm.nih.gov/Blast). The conserved motifs are boxed and the name of each motif is written in words.

**Figure 2:**
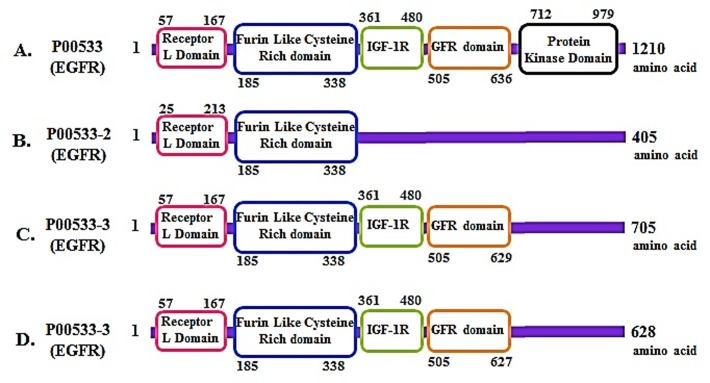
Schematic diagrams showing the domain organization in (A-D) Domain analysis was done using Scan Prosite at (http://expasy.org).

**Figure 3:**
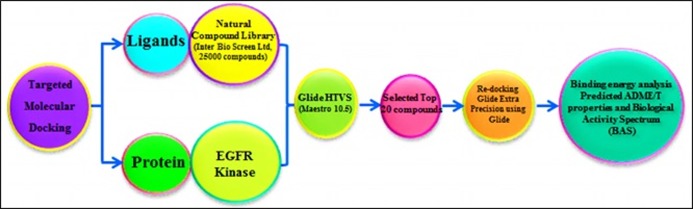
Workflow of screening of targeted compounds against EGFR kinase

An alignment of the complete amino acid sequence of EGFR and its isoform using BLAST (http://blast.ncbi.nlm.nih.gov/Blast) was completed. The protein sequence of EGFR (1210 amino acid) was aligned with the other EGFR genes sequence of, and conserved motifs are boxed in red color (Figure 1). The results indicate that EGFR contains all the specific topics including signal peptide domain, nucleotide binding sites and the protein kinase domain. Similarly, the results show that EGFR is highly conserved and it possesses all the characteristic motifs (Figure 2). 

### Molecular docking and binding energy analysis:

This examination is concentrated to investigate the firm focused on inhibitor against EGFR kinase utilizing atomic docking approach. Atomic docking of EGFR kinase against original mixes has been done. To begin with, we performed HTVS of IBS against the EGFR kinase appeared beneficial Table 1. Further, 20 best combinations from every protein atoms that have negligible Gscore sub-atomic docking were performed using the XP method of GLIDE. In the present examination, our outcome featured that aggravates a prevalent dock score for proteins EGFR kinase appeared in Table 1. Protein-ligand connections highlighted that the lipophilic, hydrogen holding, pp stacking, and cation-p communications speak to a decision commitment at the dynamic site. Sub-atomic docking activity recognizes the premier docking free vitality esteem (Gscore) against these receptor particles. Sub-atomic docking aftereffect of EGFR kinase against IBS original mixes divulged that mixes STOCK1N-98911, STOCK1N-98869, and STOCK1N-98896 yielded the best Gscore-12.22, - 8.207and - 7.061kcal/mol, individually. The atomic docking study has done seek to outline the protein - ligands communications and to condense the different securities, for example, hydrogen and electrostatic protection. 

**Figure 4:**
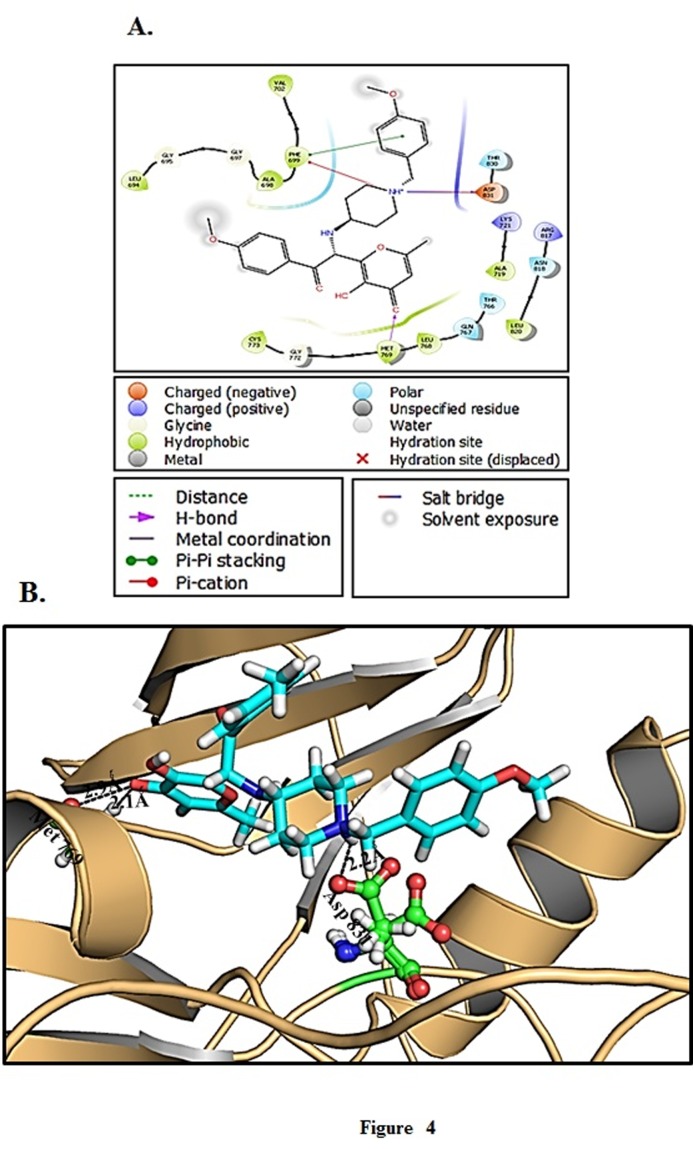
Molecular docking of compounds with EGFR kinase: (A) 2D schematic diagram showing interactions of compound STOCK1N-98911. (B) Cartoon view of EGFR kinase with compound STOCK1N-98911.

STOCK1N-98911 was observed to be most potent and pleasantly limited into the dynamic site of EGFR kinase with best Gscore contrasted with Docetaxel (Table 3). Compound STOCK1N-98911 showed three hydrogen bonds with Met: 769 and Asp: 831 of EGFR kinase 2.9 Aring;, 2.1 Aring;, and 2.2 Å separately (Figure 4B). The compound STOCK1N-98911 additionally associates with the EGFR kinase restricting site by connecting with different deposits (Ala: 696 and Phe: 699) when contrasted with Docetaxel appeared in Figure 4A. Chloroquine compound cooperates with the EGFR kinase-binding site by collaborating with deposits (Lys: 692 and Glu: 780) appeared in Table 1. Sub-atomic docking thinks about proposed that the various van der Waals, covalent, carbon-hydrogen, Pi alkyl and electrostatic collaborations are the critical power for holding of mixes STOCK1N-98911, STOCK1N-98869 and STOCK1N-98896 together with the EGFR kinase. Once more, to watch the general clearness of docking examines, we presented Prime MM-GBSA way to deal with ascertaining ligand-restricting energies. In our investigation, the coupling vitality of STOCK1N-98911, STOCK1N- 98869, and STOCK1N-98896 indicates a stable holding connection and the precision of ligand-protein official (Table 1). In this manner, at long last finished up mixes STOCK1N-98911, STOCK1N-98869 and STOCK1N-98896have demonstrated better restricting vitality for EGFR kinase, and it might be considered as an apparent inhibitor of the EGFR kinase. 

### ADME and Toxicity analysis:

Pharmacokinetic and pharmaco dynamics properties of the lead mixes were assessed by the Qikprop utility of Maestro 10.5. Mixes STOCK1N-98911, STOCK1N-98869 and STOCK1N-98896 yielded the best Score. A most intriguing part of these mixes is their commendable QPlogPo/w, QPlogHERGK+ channels, QPlogBB, QPlogKP and QPlogKhsa values that fulfill the Lipinski's Rule of Five (Table 2). The chosen properties are known to impact digestion, cell saturation, and bioavailability. All the anticipated features of the lead mixes were in the range for 95% of known oral medications and furthermore fulfilled the Lipinski's standard of five to be considered as a medication like potential. 

### Boiled-egg Plot:

**Figure 5:**
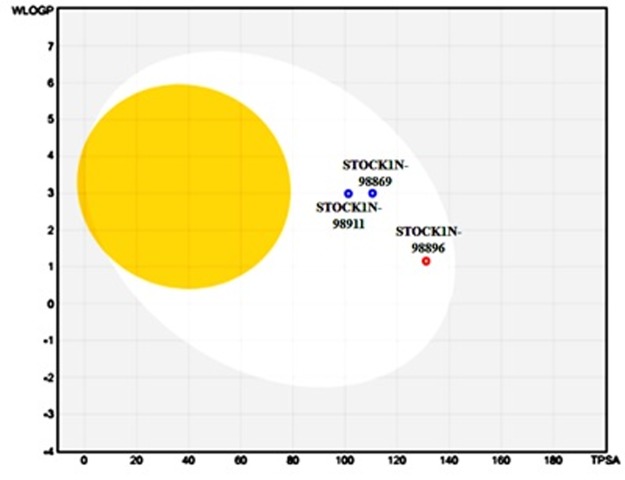
Boiled-egg Plot.

The compounds STOCK1N-98911, STOCK1N-98869, and STOCK1N-98896 were plotted in the bioled - egg plot. Table 3 abridges the consequences of the scheme. Perceptions demonstrate that every one of the three medications shows high GI assimilation and an adverse outcome for Blood-Brain pervasion. This perception legitimizes the position of all the three mixes in the white district of the boiled-Egg plot. The virtual screened tranquilize with STOCK1N-98911; STOCK1N-98869 and STOCK1N-98896 demonstrate the most astounding an incentive for TPSA and lies nearly in the focal point of the white district. None of the mixes fall in the dark locale of the plot, which affirms that every one of these mixes shows high GI ingestion and is largely BBB not penetrable (Figure 5). 

### Biological activity predictions:

Using PASS online server, selected bioactive constituents were obtained to evaluate the possible biological activity. The biological activity spectrum (BAS) of a compound is known to have pharmacological effects, specific toxicities, and mechanisms of action occurring due to compounds. Because these probabilities can be calculated independently, the Pa and Pi values vary from 0 to 1, and Pa + Pi < 1. Pa belongs to the class of active whereas Pi is for inactive compounds 22. PASS prediction results showed that the highest Pa value than Pi value come off for anticancer activity and hence indicated the anticancer of selected compounds (Table 4). However, all compounds have shown a significant Pa value as compared to Pi value. These compounds might be inhibiting cancer infection via blocking EGFR kinase action as evidenced by docking studies. 

## Conclusion

From the perspectives of docetaxel resistant ovarian cancer, the current study is an attempt to explore out the therapeutic potentiality of STOCK1N-98911, STOCK1N-98869 and STOCK1N- 98896 compound. The compound STOCK1N-98911, STOCK1N- 98869, and STOCK1N-98896 shows a high degree of binding to the EGFR kinase. The pharmacophore mapping of the molecule shows the efficiency with which it binds to the receptor structure. The ADMET profile of this ligand is highly favorable, which predicts the ligand would give positive results when in vitro and in vivo studies are conducted. Furthermore, the review is also useful for further clinical based studies and also for the validation of toxicological and pharmacokinetic study. 

## Conflict of Interest

Authors declare no conflict of interest.

## Figures and Tables

**Table 1:** Lowest binding energy for the Ligands-EGFR kinase interaction, along with scores for various interaction types, as detected by GLIDE

Compounds ID	Binding Energy	GScore	Lipophilic E vdw	H-bond	Electro	Protein ligands interaction
	MM-GBSA (kcal/mol)					
STOCK1N-98911	-62.7277	-12.22	-3.436	-1.025	-2.65	Ala:696, Phe:699, Met:769 and Asp:831
STOCK1N-98869	-51.2494	-8.207	-4.631	-1.032	-0.428	Lys:721and Asp:831
STOCK1N-98896	-49.2545	-7.061	-3.708	-1.805	-0.669	Met:769 and Gln:767
Known Inhibitor						
Docetaxel	-47.1282	-5.14	-4.532	-1.072	-0.931	Lys:692 and Glu:780

**Table 2:** Evaluation of drug-like properties of the lead molecules by Qikprop Maestro 10.5 molecular docking suite

Molecule	QPlog Po/w	Q P log	QPP Caco	Q P log	QPP	Q Plog
	(-2.0 to 6.5)	HERG	(nm/s)	BB	MDCK	Kp
		(acceptable range:	<25 - poor	(-3 to 1.2)	(nm/s)	(-8.0 to -0.1)
		above -5.0)	>500 - great			
STOCK1N-98911	3.09	-8.158	50.367	-0.543	23.953	-5.767
STOCK1N-98869	3.198	-8.015	50.441	-0.628	23.987	-5.864
STOCK1N-98896	0.887	-4.804	100.053	-1.541	41.088	-4.179

**Table 3:** Boiled egg parameters

Molecule	MW	TPSA	XLOGP3	MLOGP	GI absorption	BBB permeant
STOCK1N-98911	492.56	101.24	3.37	0.58	High	No
STOCK1N-98869	522.59	110.47	3.34	0.3	High	No
STOCK1N-98896	378.34	131.08	0.58	0.93	High	No

**Table 4:** Biological activity spectrum of compounds (Pa � Active; Pi � Inactive)

Molecule	Pa	Pi	Activity
STOCK1N-98911	0.919	0.049	Anticancer
STOCK1N-98869	0.812	0.042	Anticancer
STOCK1N-98896	0.921	0.018	Anticancer
